# Validation of a Novel, Flash‐Freezing Method: Aluminum Platform

**DOI:** 10.1002/cpet.46

**Published:** 2020-11-25

**Authors:** Ahmet Imrali, Christine S. Hughes, Abigail S. Coetzee, Francesca R. Delvecchio, Amina Saad, Rhiannon Roberts, Claude Chelala, Jo‐Anne ChinAleong, Hemant M. Kocher

**Affiliations:** ^1^ Pancreatic Cancer Research Fund Tissue Bank (PCRFTB) London United Kingdom; ^2^ Centre for Tumour Biology, Barts Cancer Institute—A CR‐UK Centre of Excellence Queen Mary University of London Charterhouse Square London United Kingdom; ^3^ Barts and the London HPB Centre, Department of Surgery and Pathology, Barts Health NHS Trust The Royal London Hospital London United Kingdom

**Keywords:** DNA, fresh tissue, immunofluorescence, liver, pancreas, RNA

## Abstract

Stored biological materials should have minimal pre‐analytical variations in order to provide researchers with high‐quality samples that will give reliable and reproducible results, yet methods of storage should be easy to implement, with minimal cost and health hazard. Frozen tissue samples are a valuable biological resource. Here we compare different methods, such as liquid nitrogen (LN) or dry ice (DI), to a cheap and safe alternative using an aluminum platform (AP). Murine fresh liver and pancreas tissues were used with varying lengths of warm ischemia time. Quality assessment was based on histological evaluation, DNA and RNA extraction and quantification, and RNA degradation analysis, as well preservation of antigens for immunofluorescence, in a blinded manner. Both in superficial and deep tissue sections, based on histological assessment, AP is superior to DI, or as good as LN techniques in terms of presence of ice crystals, cutting artifacts, and overall quality/structural preservation. DNA and RNA were successfully extracted in reasonable quantities from all freezing techniques, but RNA degradation was seen for pancreas samples across all techniques. Immunofluorescence with cytokeratin8 (CK‐8), alpha smooth muscle actin (αSMA), CD3, and B220 shows equally good outcomes for AP and LN, which are better than DI. The aluminum platform is a cheap, yet reliable method to freeze samples, rapidly preserving histological, antigenic, and DNA/RNA quality. Wider testing is required across different sample types. © 2020 The Authors.

**Basic Protocol**: Flash‐freezing fresh tissue with aluminum platform

**Alternate Protocol 1**: Freezing fresh tissue with liquid nitrogen

**Alternate Protocol 2**: Freezing fresh tissue with dry ice

## OVERVIEW AND PRINCIPLES

Variation in biological sample collection and preservation can lead to a large proportion of pre‐analytical errors (∼30%‐75%) in laboratory research (Betsou et al., 2009; Bonini, Plebani, Ceriotti, & Rubboli, 2002). One of the most important resources for research is fresh tissue collected from surgery and other sampling procedures. However, it is not always possible to immediately deliver the fresh tissue from these clinical arenas to researchers. Therefore, rapid freezing is widely considered a suitable method for tissue preservation as an interim solution, usually within 20 min of removal from the host (Shabihkhani et al., 2014). Frozen tissue is the favored bio‐specimen for modern testing because it produces a higher yield and quality of nucleic acids and proteins than the more common formalin‐fixed paraffin‐embedded (FFPE) tissue (Tang, Hu, Muallem, & Gulley, 2012). Until now, the collection of frozen bio‐specimens has mostly been used for research programs, but “next‐generation” testing is moving rapidly into daily clinical care, suggesting that frozen tissue collection may become routine for diagnostics when cancer or certain disorders are suspected. Consequently, it is anticipated that pathology departments and biobanks will have to store and disseminate increasing numbers of frozen bio‐specimens (Balarajah et al., 2016).

**Figure 1:**
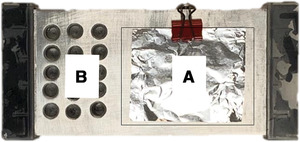
The aluminum platform (AP) developed at Pancreatic Cancer Research Fund Tissue Bank (PCRFTB). The platform is composed of a flat surface (Part A) where fresh tissue is frozen by cryogenic spray. The frozen tissue is kept cold on the platform until it is transferred into the pre‐cooled tubes, located in the tube‐holding part (Part B) of the aluminum platform (see Basic Protocol). Use of the AP is compared with use of liquid nitrogen (LN) and dry ice (DI) in Alternate Protocols 1 and 2, respectively.

**Figure 2:**
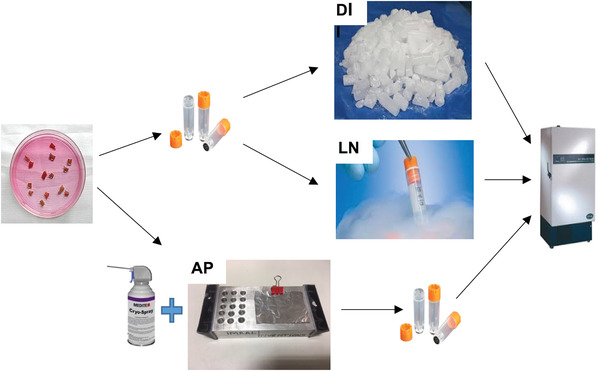
Schematic representation of the three freezing methods used in this article: aluminum platform (AP), dry ice (DI), and liquid nitrogen (LN). The fresh tissue specimen is placed in a FluidX™ tube, the lid is closed firmly, and the tube is either placed in dry ice or briefly immersed in liquid‐phase nitrogen for freezing. In the AP freezing method, the fresh tissue specimen is placed on the pre‐cooled AP, covered with aluminum foil, and frozen with cryogenic spray before the frozen tissue is placed into the FluidX™ tubes. Finally the frozen tissues are transferred to –80°C freezer for long‐term storage before analysis.

Liquid nitrogen (LN) freezing is considered a gold‐standard technique for rapid freezing of biological samples, since at −197°C, the tissue specimen will freeze instantly. This minimizes the risk of ice‐crystal formation and consequential tissue artifacts, although the ‘Leidenfrost effect’ (defined in Troubleshooting) has been reported in some samples (Rivas Leonel, Lucci, & Amorim, 2019). However, LN poses a biohazard and is rarely used in clinical areas (Mehta, Baranova, & Birerdinc, 2012). Consequently, dry ice (DI; –80°C) is used as an interim measure in clinical areas with the aim of freezing rapidly once the sample is outside of the host, but it has number of drawbacks including depth of freezing and possible ice‐crystal formation due to slower sample freezing than LN. In order to rapidly, fully, and safely freeze samples in a clinical setting, we developed a modification of the DI method using a combination of aluminum platform (AP; Fig. 1) and cryogenic spray to enable rapid freezing and improved preservation of frozen tissue in a cheap, reliable manner (Fig. 2). We present the findings of our initial analyses from murine samples.

Note that the protocols below are written for a normal murine tissue (liver and pancreas were tested) that does not represent any specific disease. Refer to any model‐specific details provided by the source of the model (e.g., a biobank), and adjust parameters accordingly. Steps containing potential critical parameters are noted in the protocols below.

Please read the entirety of the protocols before proceeding with culture. All protocols should be performed under aseptic culture conditions using sterile supplies and cell culture−grade reagents, and wearing appropriate personal protective equipment (PPE). Use good laboratory practices at all times while working in the laboratory. Refer to the Material Safety Data Sheet (MSDS) for all reagents prior to use for important safety information. Refer to the Certificate of Analysis or other documentation accompanying the material, to ensure it has been verified to be free of contamination. Treat all human‐derived material as potentially biohazardous and handle under the appropriate biosafety conditions. Refer to U.S. Public Health Service Guidelines (see Internet Resources) and your institution's safety office for more information.

## STRATEGIC QUESTIONS

#### Can I use these protocols with any type of tissue?

Yes. In our rigorous analysis we used murine liver and pancreas tissue samples, since these tissue specimens are among the most sensitive to tissue degradation and ice‐crystal formation if not handled properly. However, any other type of tissue can be used in the protocols described below. We recommend thorough testing before routine use.

#### Do I need special training for any of the protocols described?

Yes: for the LN protocol, due to the health risks involved in handling liquid nitrogen, special training is required. However, the dry ice and cryospray/aluminum platform methods have almost no health risks. Therefore, no special training is required to carry out those techniques.

A schematic representation of all three freezing methods used in this article is outlined in Figure 2.

#### What should I do if I don't have immediate access to a long‐term storage freezer when I freeze the fresh tissue specimen?

If immediate access to a long‐term storage freezer is not possible, or if frozen samples need to be transported to the freezer, they can be stored in dry ice to ensure that the samples never reach thawing temperature (ideally never above −20°C). The temperature of dry ice is around −79°C. Ensure that enough dry ice is present, as dry ice evaporates (via sublimation) with time.

#### Will the tissue architecture be preserved?

Students can carry out in‐house assessment of tissue architecture, as we have performed. Briefly, hematoxylin and eosin (H&E) staining was performed on all frozen and cryosectioned slides with a standard staining protocol carried out using a LEICA CV5030 Autostainer XL with Gill III hematoxylin (Merck, KGaA, HX84908574). A senior pathologist examined the H&E‐stained frozen sections in a blinded manner for sample quality/preservation and the level of ice‐crystal formation. Five grades described this as: 10 (unsatisfactory), 8 (of limited use), 6 (Fair/useable), 4 (Good), 2 (Very good) and 0 (Excellent). The formation of ice crystals was also categorized into four grades, described as: 0 (None), 2.5 (Mild), 5 (Moderate), and 8 (Severe). The Kruskal‐Wallis non‐parametric analysis of variance, *p* < 0.05 test was used to compare the three freezing techniques in GraphPad Prism8™.

**Figure 3:**
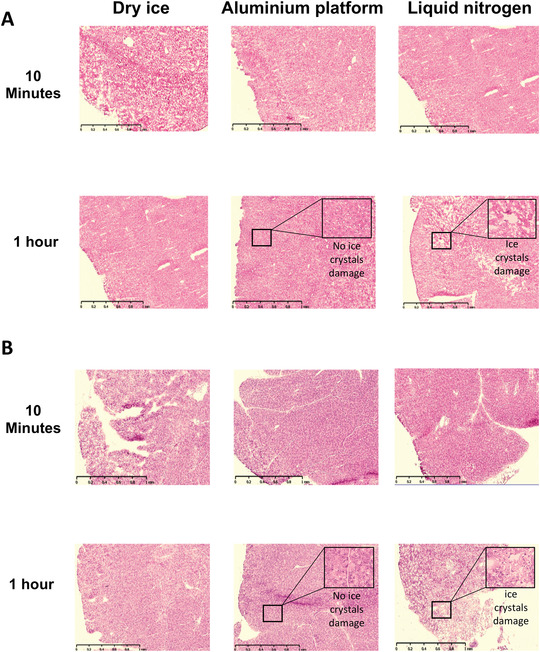
Representative images of low‐power microscopy of H&E‐stained cryosections are shown for the three different freezing techniques. Normal mouse liver (**A**) and pancreas (**B**) at 10‐min and 1‐hr time points during which the fresh tissue is placed in PBS at room temperature (warm ischemia) before freezing. Scale bar: 1 mm.

**Figure 4:**
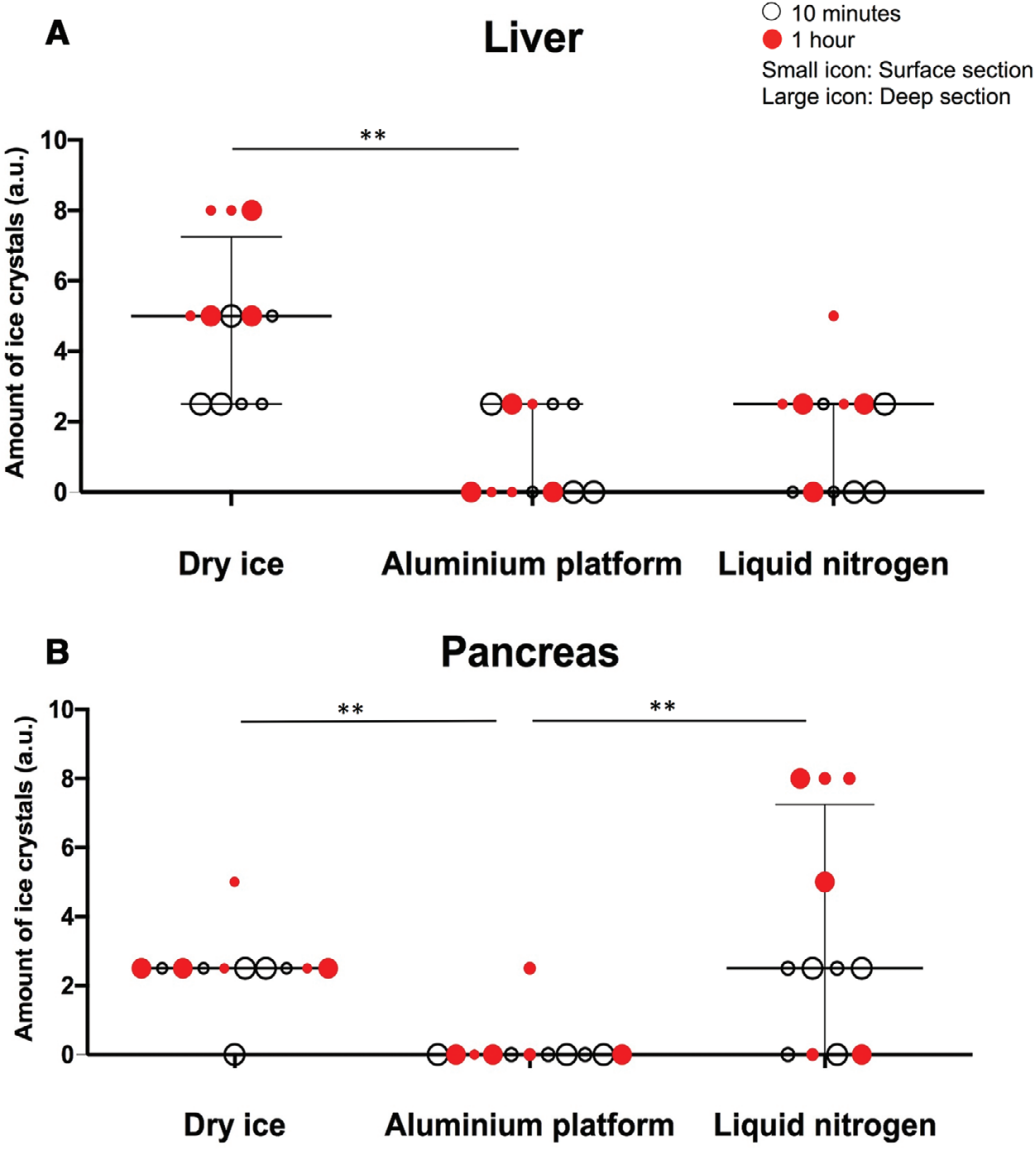
The amount of ice‐crystal formation (arbitrary units) across all freezing techniques. Fresh‐frozen mouse normal liver (**A**) and pancreas (**B**). Summary data shown as median with interquartile range. Kruskal‐Wallis test was used for statistical analysis.

**Figure 5:**
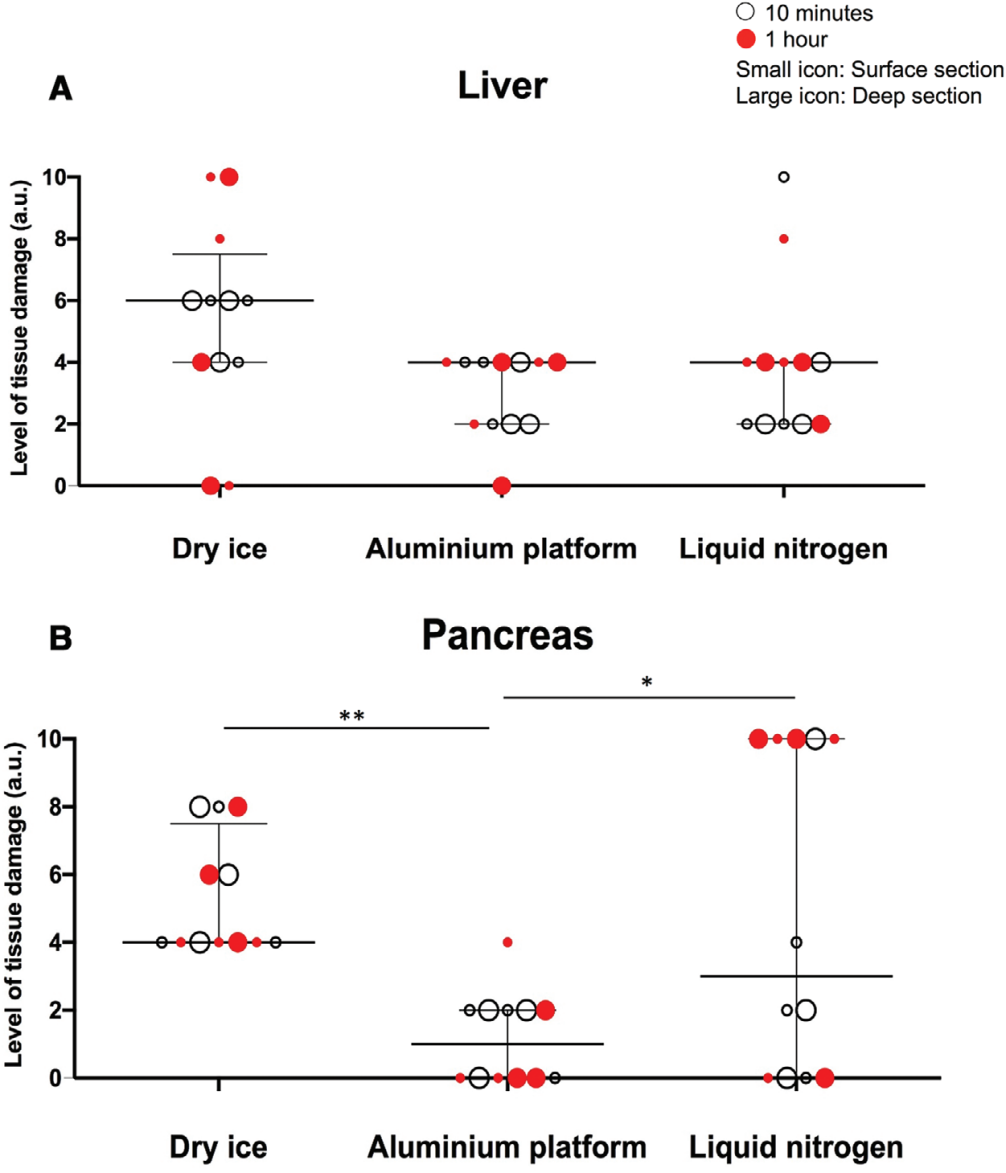
The level of tissue damage for each freezing method, using liver (**A**) and pancreas (**B**) tissues. The tissue damage is validated as an arbitrary unit, considering the overall tissue quality, including: autolysis, cutting artifacts, and the extensive damage caused as a result of ice crystals. Kruskal‐Wallis test was used for statistical analysis.

Upon blinded H&E slide evaluation for tissue preservation (Fig. 3), it was evident that the amount of ice‐crystal formation resulting from the AP technique was significantly lower than that from the DI alone, and was comparable to LN for liver tissues (Fig. 4A). For pancreatic tissue, the AP technique was significantly better than both the DI and LN techniques (Fig. 4B). The preservation of the liver tissue architecture was comparable between the three techniques (Fig. 5A), but the pancreas was best preserved with the AP technique (Fig. 5B).

#### Will the DNA and RNA be preserved?

Students can carry out in‐house assessment for DNA and RNA quantity and quality, as we have performed. A Qiagen AllPrep DNA/RNA mini kit (Qiagen, 80204) was used for RNA and DNA extraction according to the manufacturer's protocol. Fresh‐frozen sections of liver and pancreas tissue on slides were kept on dry ice until the RLT buffer [included in the abovementioned kit and freshly supplemented with β‐mercaptoethanol (β‐ME), as per manufacturer's protocol] was added to the tissue. Extracted DNA and RNA samples were quantified using the Nanodrop‐One spectrophotometer (Thermo Fisher Scientific) per the manufacturer's protocol. The quality of RNA was measured using the Agilent 2100 Bioanalyzer instrument with RNA 600 Pico chip by Bart's and the London Genome Centre (Blizard Institute, London) per the manufacturer's protocol.

**Figure 6:**
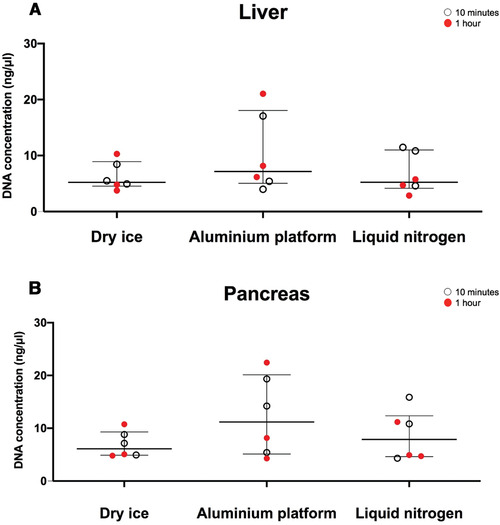
The concentration of DNA extracted from fresh‐frozen mouse liver (**A**) and pancreas (**B**) tissue sections. DNA extraction was carried out using Qiagen AllPrep DNA/RNA mini kit. The tissue‐lysis step, which causes rapid deactivation of DNase and RNase, was done on the frozen tissue sections on dry ice to minimize the effect of endogenous DNase and RNase. All sections used were deeper cut sections.

**Figure 7:**
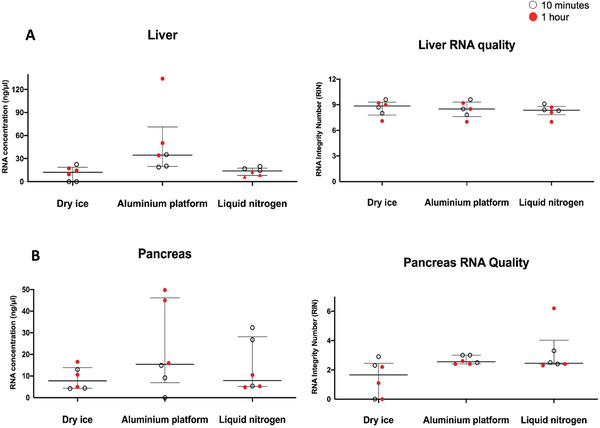
The concentration of extracted RNA from liver (**A**) and pancreas (**B**) fresh‐frozen tissue sections. The extracted RNA samples were also tested for the RNA Integrity Number (RIN), which represents the level of preservation, and hence the quality of the RNA.

A good quantity of DNA was extracted from frozen sections across all three freezing techniques and both tissue types (Fig. 6). While RNA quantity and quality were similar between the three techniques, extraction from the pancreas tissue uniformly yielded poorer‐quality RNA across all techniques, compared to liver tissue (Fig. 7).

#### Will the antigenicity for immunostaining be preserved?

Students can carry out in‐house assessment for antigen preservation, as we have performed.

For α‐SMA and CK8 double‐staining, the sections were dried at room temperature and then fixed in 4% formaldehyde (20 min), washed three times, each time for 5 min in PBS‐Tween (0.05%), blocked with 5% goat serum in PBS for 1 hr at room temperature, and incubated with FITC‐conjugated mouse monoclonal anti‐α‐SMA antibody (Abcam, ab8211, 1:500) as well as rabbit monoclonal anti‐CK8 antibody (Dako, M3652, 1:100) overnight, at 4°C. After three 5‐min washes in PBS‐Tween (0.05%), AlexaFluor^®^ 546−conjugated goat anti‐rabbit IgG (Thermo Fisher Scientific, A‐11035, 1:500) was used to incubate slides at room temperature for 1 hr. After three further 5‐min washes in PBS‐Tween (0.05%), nuclei were counterstained with anti‐fade mounting medium containing DAPI (Molecular Probes, S36938).

For B220 and CD3 double‐staining, after fixation, washing, and blocking as described above, incubation took place with rat monoclonal anti‐B220 antibody (BD Bioscience, 550286, 1:50) as well as rabbit monoclonal anti‐CD3 antibody (Abcam, ab5690, 1:50) overnight at 4°C. Following washing and incubation with AlexaFluor^®^ 546−conjugated goat anti‐rat IgG (Thermo Fisher Scientific, A‐11081, 1:400) and AlexaFluor^®^ 488−conjugated goat anti‐rabbit IgG (Thermo Fisher Scientific, A‐32731, 1:400) at room temperature for 1 hr, the nuclear counterstaining was performed as described above. All sections were scanned using a slide scanner (NanoZoomer 60 SQ; Hamamatsu Photonics).

**Figure 8:**
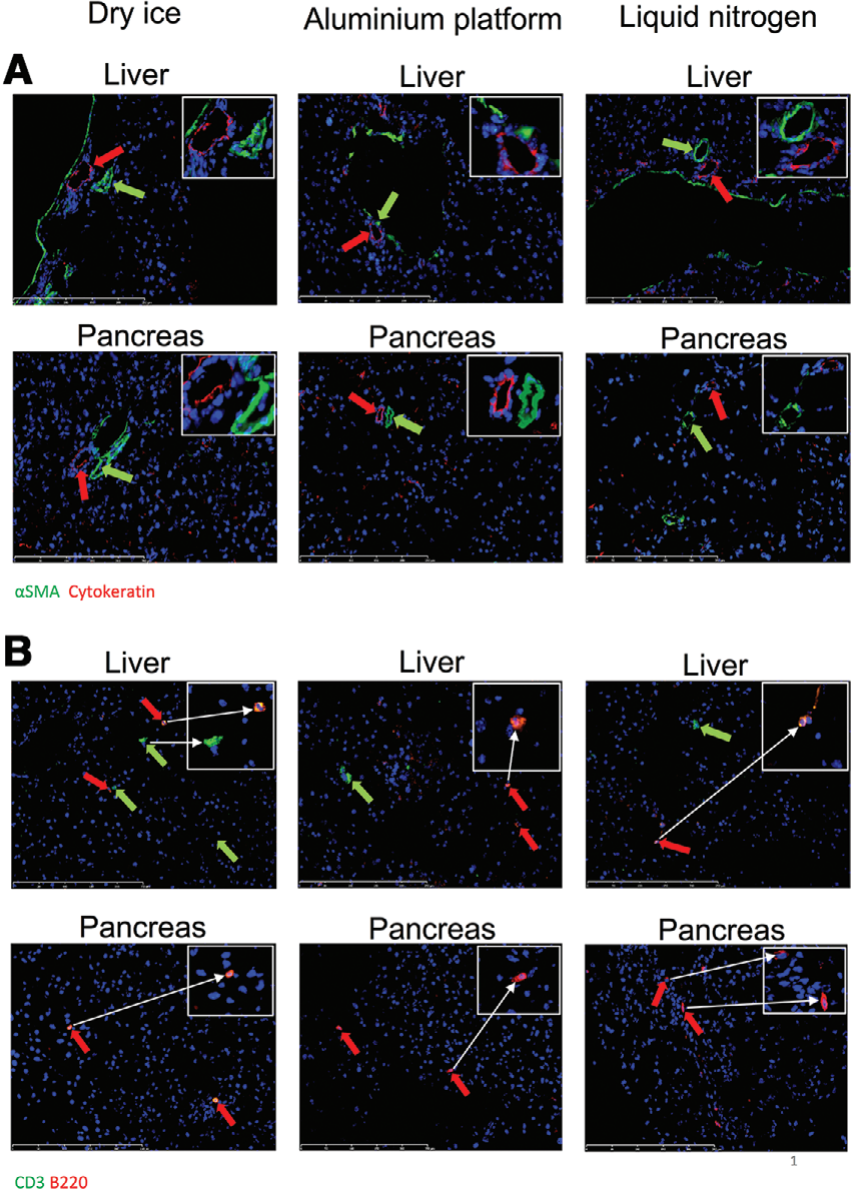
Immunofluorescent (IF) staining of the fresh‐frozen sections. Both mouse liver and mouse pancreas sections were IF stained with αSMA (green), CK‐8 (red) (Panel A, scale bar: 250 µm), as well as B220 (red) and CD3 (green) (Panel B, scale bar: 250 µm) antibodies. Images obtained indicated positive staining for all antibodies across all three freezing techniques.

Good‐quality immunofluorescence (IF) staining could be obtained across all techniques in tissues for epithelial cells (CK8), fibroblasts/pericytes (α‐SMA), T cells (CD3), and B cells (B220), though staining in the pancreas uniformly showed very scarce T and B cells (Fig. 8). Qualitatively, the AP technique performed as well as LN, and better than DI.

## STRATEGIC PLANNING

Before performing the protocols based on the technique of freezing, make sure all of the required equipment and consumables are available and ready. For example, for the AP method (Basic Protocol), the aluminum platform needs to be placed on dry ice or kept in –80°C freezer for at least 10 min to let it cool down, and during this procedure the aluminum platform should always sit on dry ice in order to prevent it from warming up. When cooling the aluminum platform on dry ice, make sure that enough dry ice is placed in an insulated container with a large enough surface area to accommodate the aluminum platform (20 cm × 10 cm).

For the LN technique (Alternate Protocol 1), the liquid nitrogen should be obtained from the source and transported according to the safety rules for the transportation of hazardous materials; a risk assessment form should be filled out in case of accidental spillage of liquid nitrogen during transport, and the liquid nitrogen should be placed on a safe platform (uncluttered bench) in a Dewar (special transport flask for liquid nitrogen).

For the DI method (Alternate Protocol 2), make sure that enough dry ice is placed in an insulated container with a large enough surface area to accommodate the cryovials.

The transport time for the tissue specimens obtained from a source (e.g., mouse, human) should be minimized in order to avoid degradation due to tissue autolysis. If different time points for the experiment are planned (for example, we tested at 10 min and 1 hr time points in validation of the AP method), then the tissue reserved for later time points should be placed in phosphate‐buffered saline (PBS) solution to prevent it from drying out during incubation at room temperature.

## SAFETY CONSIDERATIONS

Appropriate personal protective equipment (PPE) should be worn (at least gloves, goggles, and lab coat). Specifically for liquid nitrogen, additional PPE is required; these are listed in the materials section of the LN protocol. Handling of the liquid nitrogen requires specific training due to its high potential risk to health and safety. Make sure that the appropriate training is obtained on how to handle and work with liquid nitrogen, or at least training on the handling, management, and disposal of laboratory hazardous materials, chemicals. and waste generally. Also, the user of any of the techniques in this protocol should know the first aid measures for chemical burns and frostbite, as these freezing techniques are likely to cause frostbite if not carried out correctly.

Refer to the Material Safety Data Sheet (MSDS) for all reagents prior to use, for important safety information. Refer to the Certificate of Analysis or other documentation accompanying the material, to ensure it has been verified to be free of contamination. Treat all human‐derived material as potentially biohazardous, and handle under the appropriate biosafety conditions. Refer to U.S. Public Health Service Guidelines (see Internet Resources) and your institution's safety office for more information.

## FLASH‐FREEZING FRESH TISSUE WITH ALUMINUM PLATFORM (AP)

This protocol describes the detailed steps for flash‐freezing fresh tissue specimens using our novel cryospray and aluminum platform (AP) method. The aluminum platform (Fig. 1) is specifically designed and manufactured by Imrali Inventions Ltd. for the Pancreatic Cancer Research Fund Tissue Bank (PCRFTB), London. The platform is composed of a flat surface (Part A) and tube‐holding holes (Part B). Cryospray is used for rapidly freezing fresh tissue on the aluminum platform, while the aluminum platform keeps the tubes and the frozen tissue cold and prevents any thawing during the procedure.

*CAUTION*: Please take extra care when using cryospray and avoid spraying it directly onto fingers, as prolonged direct spraying (>5 s) onto fingers can cause serious frostbite (injury caused by freezing of the skin and underlying tissue).

The specifications of the aluminum platform are:

Dimensions: 15 cm × 8 cm × 3 cm (L × W × D),
15 holes having 8 mm diameter and 20 mm depth in 3 × 5 matrix format on the left‐hand side of the platform.

This platform can easily be manufactured from an aluminum block by drilling out the holes on the left‐hand side as specified above using 8‐mm HSS drill bit in a local machine shop. The sides of the platform should have plastic handles screwed into the platform to allow safe handling when cold, and a small paper clip screwed in the middle of the flat surface of the platform for easily holding the aluminum foil sheet in place, in order to prevent it from being blown away when sprayed with cryospray

If this is not possible, then a special request for manufacturing for purchase of the aluminum platform can be made by e‐mailing Imrali Inventions Ltd. on their website (https://www.imraliinventions.com) in the Contact Us section.

### Materials

Dry ice
Fresh tissue samples (collected from surgery or any other relevant source)
Phosphate‐buffered saline (PBS; Sigma‐Aldrich, cat. no D8537‐500ML; without calcium chloride and magnesium chloride; liquid, sterile‐filtered, suitable for cell culture)
Cryospray (Cellpath™ Cryospray Freezer Spray; Fisher Scientific, cat. no: 12705308)

Goggles and other appropriate PPE (see Safety Considerations)
Insulated dry ice box
Aluminum platform (see introduction to this Basic Protocol)
Petri dish (or similar sterile tissue container)
Scalpel
0.7‐ml FluidX™ Tubes
Sample labels
Pre‐cut aluminum foil sheets (5 cm × 4 cm)
Tweezers

**Figure 9:**
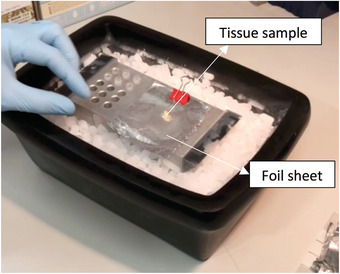
Image showing the tissue specimen placed in the middle of an aluminum foil sheet that is clipped onto the pre‐cooled aluminum platform.

**Figure 10:**
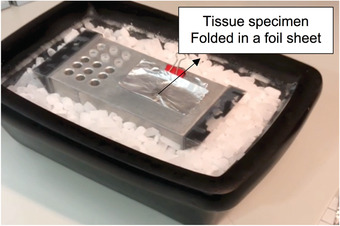
Image showing the foil sheet folded in the middle section to cover the tissue specimen while allowing the tissue specimen to be in contact with the surface of the aluminum platform. Covering fresh tissue with an aluminum sheet clipped to the platform stabilizes the tissue and prevents it from being blown away and out of the aluminum platform when sprayed with cryospray.

1 Place some dry ice into an insulated dry ice container having a minimum open surface area larger than that of the aluminum platform (20 cm × 10 cm).
2 Place the aluminum platform (AP) on dry ice for at least 10 min to allow it to cool down. Alternatively, the AP can be placed in –80°C freezer for at least 10 min.
3 Obtain the fresh tissue samples and place them in an appropriate container (e.g., petri dish) in PBS at room temperature during transport to the lab where the aluminum platform is set up.
  Make sure the size of the tissue to be frozen is not larger than the size of the 0.7‐ml FluidX™ tube. If the tissue is too large, cut it into a smaller, appropriate size (5 mm × 3 mm × 3 mm) using the scalpel.
4 Label FluidX tubes and place them into the tube‐holding part of the aluminum platform (Fig. 1B) to cool them down.
5 Take a piece of pre‐cut foil sheet (5 cm × 4 cm) and place it onto the flat surface of the AP (Fig. 1A).
6 Take the appropriate‐sized fresh tissue sample (5 mm × 3 mm × 3 mm) so that it can fit in the FluidX tube after freezing and place it into the middle of the aluminum foil sheet using tweezers (Fig. 9). Fold the foil sheet from just below the tissue specimen, covering the tissue with the aluminum sheet (Fig. 10).

**Figure 11:**
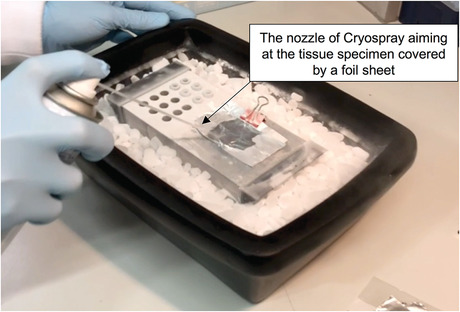
Image showing the tissue folded in aluminum sheet being sprayed with cryospray for freezing. The cryospray nozzle should be aimed and sprayed directly onto the tissue during freezing.

7 Spray the folded aluminum foil with cryospray, aiming at the place where the tissue specimen is covered by the aluminum foil sheet. Continuously spray for 10 s (Fig. 11).

8 Gently unfold (open) the aluminum sheet with the help of tweezers, making sure the tissue covered by the aluminum sheet is always pushed down and in contact with the flat surface of the AP.
  At this point, the tissue is frozen rock solid; it may also be stuck to the aluminum foil due to the moisture on the surface of the tissue when in contact with the aluminum foil, which can form a freeze‐bond during freezing.
9 Very carefully and gently remove the frozen tissue with tweezers: it might be stuck to the aluminum foil. If stuck, gently nudge the tissue with tweezers to free it from the aluminum foil and immediately place it into the appropriately labeled pre‐cooled tube in the tube‐holding part of the AP.
10 Close the lid of the tube and keep it in the AP until all tissue specimens are frozen.
11 Repeat steps 1 to 10 for the remaining tissue specimens.
12 Keep AP on dry ice and transfer to a long‐term storage freezer at –80°C, where the tubes can now be removed from the AP tube‐holding positions into tube racks and placed into the freezer.

## Alternate Protocol 1 FREEZING FRESH TISSUE WITH LIQUID NITROGEN (LN)

This protocol describes detailed steps for freezing fresh tissue specimens using liquid nitrogen, a process that is carried out at an extremely low temperature (–196°C). At this low temperature, the freezing of the tissue occurs more rapidly than with any other method; thus, liquid nitrogen is considered to be the gold‐standard method for freezing fresh tissue. However, due to the potential safety hazards involved in using liquid nitrogen, special training for handling and disposing of liquid nitrogen should be obtained from the appropriate accredited training body. Please do not handle liquid nitrogen without appropriate training. The necessary PPE to be worn during this procedure are at least latex disposable gloves together with cryogenic gloves for better protection, face shield, lab coat, and oxygen monitor. Liquid nitrogen can dilute the air in a poorly ventilated space and reduce the oxygen levels, causing hypoxia; therefore, this procedure should be carried out in a well‐ventilated lab facility room where oxygen levels are monitored and linked to a low‐oxygen detection/warning system. Make sure liquid nitrogen is stored and transported in an insulated cylindrical Dewar, and that the lab area where this Dewar is located is free of clutter, to reduce the risk of accidental spillage. During this procedure, the user should expect rapid boiling of the liquid N_2_ around the sample tube upon insertion of the tube into the liquid nitrogen. This boiling reduces and disappears as the tube cools down and reaches the boiling temperature of LN.

### Materials

Liquid nitrogen
Dry ice
Fresh tissue samples (collected from surgery or any other relevant source)
Phosphate‐buffered saline (PBS; Sigma‐Aldrich, cat. no D8537‐500ML; without calcium chloride and magnesium chloride; liquid, sterile‐filtered, suitable for cell culture)

Goggles
Cryogenic gloves (specifically required PPE for handling liquid nitrogen)
Face shield (specifically required PPE for handling liquid nitrogen)
Oxygen monitor (specifically required PPE for handling liquid nitrogen)
Cylindrical Dewar (Universal‐Style Stainless Steel and Glass Dewar Flask with Handle and Lid, 2 L; Cole‐Parmer, cat. no UY‐03774‐14; available in 1‐L, 2‐L, 4.5‐L, and 7‐L sizes from the supplier)
Insulated dry ice container
0.7‐ml FluidX™ Tubes [compatible with short‐term (5‐10 min) exposure to liquid‐phase nitrogen]
Sample labels
Petri dish (or similar sterile tissue container)
Tweezers (normal size) and long tweezers (minimum 20 cm long)

1 Liquid nitrogen is delivered by the industrial gas supply company (e.g., BOC) and stored in large storage tanks. Transfer some (about 1 L) liquid nitrogen from the storage tank into a closed and insulated cylindrical Dewar and transport to the lab bench where the freezing protocol will be carried out.
2 Place some dry ice into an insulated dry ice container and place the labeled FluidX the labeled FluidX tubes in dry ice to cool them down.
3 Obtain the fresh tissue samples and place them in an appropriate container (Petri dish) in PBS at room temperature during transport to the lab where the liquid nitrogen and dry ice box are set up.
4 Place tissue specimens in appropriate pre‐labeled and pre‐cooled tubes and screw the lids on tightly.
5 Hold each tube with long metal tweezers and dip it into liquid nitrogen. Keep the tube submerged in liquid nitrogen for 30 s.
  You will hear a boiling and hissing sound coming from the tube. This is normal, as the boiling temperature of liquid nitrogen is very low (–196°C), and when in contact with a much warmer sample tube, it immediately starts to boil around the tube. As the tube cools down, this boiling reduces and stops.
6 Remove the frozen tube with the long metal tweezers and place in dry ice to prevent thawing of the sample.
7 Repeat steps 4 to 6 for the remaining samples.
8 Keep sample tubes on dry ice until transported to a –80°C freezer for long‐term storage.

## Alternate Protocol 2 FREEZING FRESH TISSUE WITH DRY ICE (DI)

This protocol describes the detailed steps for freezing fresh tissue specimens using dry ice. This is not the best technique for freezing tissue specimens, because dry ice has a relatively higher temperature of –80°C compared to liquid nitrogen's –196°C. However, using dry ice is much safer both in laboratory and clinical settings, and the results of freezing are acceptable if the steps are carried out correctly. Dry ice, just like liquid nitrogen, has potential asphyxiation properties because it sublimes directly into CO_2_ gas and results in the reduction of oxygen in a closed environment. Therefore, this protocol needs to be carried out in a well‐ventilated lab area, and, if possible, an oxygen level monitor should be used as an additional safety measure.

### Materials

Dry ice
Fresh tissue samples (collected from surgery or any other relevant source)
Phosphate‐buffered saline (PBS; Sigma‐Aldrich, cat. no D8537‐500ML; without calcium chloride and magnesium chloride; liquid, sterile‐filtered, suitable for cell culture)

Goggles
Oxygen monitor (recommended PPE for working with dry ice)
Insulated dry ice box
Petri dish (or similar sterile tissue container)
0.7‐ml FluidX™ Tubes
Sample labels

1 Collect dry ice and transport in an insulated dry ice box to the lab bench where the freezing protocol will be carried out. Place the labeled FluidX tubes in dry ice to cool them down.
  CAUTION: Make sure this area is well ventilated, as dry ice sublimes to CO_2_, reducing the levels of oxygen, and in extreme cases may cause asphyxiation.
2 Obtain the fresh tissue samples and place them in an appropriate container (Petri dish) in PBS at room temperature during transport to the lab where the liquid nitrogen and dry ice box are set up.
3 Place tissue specimens in labeled and pre‐cooled FluidX tubes (from step 1) and close the lids.
4 Place the tube in dry ice. Make sure the tube is fully inserted in the dry ice pellets and that the whole surface of the tube is covered by dry ice.
5 Keep tube in dry ice for 10 min to freeze the tissue.
6 Leave tube in dry ice and move on to the next sample.
7 Repeat from step 3 to 6 for the remaining samples.
8 Keep sample tubes in dry ice until transported to a –80°C freezer for long‐term storage.

## COMMENTARY

### Background Information

In brief, using murine liver and pancreas tissues for varying lengths of warm ischemia time (when the tissue is out of the body and not connected to blood supply as well as not immersed in ice) to mimic clinical scenarios, we have demonstrated that the novel AP technique provides as good, if not better, quality of tissue preservation in all aspects compared to LN, and better than DI alone. This is important since the gold‐standard rapid‐freezing method (liquid nitrogen) is biohazardous and has problems associated with storage, transport, and use in close, crowded clinical areas such as operating theaters and wards with multiple personnel and patients present (Mehta et al., 2012). Furthermore, the adaptation of this new freezing technique (AP) using easily portable and re‐usable materials, including DI and the AP, enables wide‐scale implementation in low‐cost environments, thus facilitating widespread tissue collection. Further testing and refinement of this technique would lead to use in clinical and research areas including biobanking.

Using a well‐validated system, such as the one described here, would enable researchers to gain confidence in tissue acquisition with fewer pre‐analytical variables. Indeed, sample handling, i.e., collection, processing, storage, and retrieval, must be conducted under validated standard operating procedures (SOPs) with critical quality assurance (QA) and quality control (QC) programs to reduce diagnostic errors (Bertschinger & Bollinger, 1995), as well as to improve biobanking standards (Balarajah et al., 2016). One of the detrimental effects of freezing fresh tissue specimens is the formation of the large ice crystals as a result of slow freezing of the tissue. This physical property of water molecules whereby large ice crystals are formed during slow freezing can affect the quality of fresh‐frozen tissue samples. Therefore, rapid freezing of fresh tissue is necessary in order to eliminate the formation of larger ice crystals. The main effect of large ice crystals becomes evident when the fresh‐frozen tissue is thawed for various experimental reasons, such as staining for specific molecular markers or histological examination of the tissue structure. The large ice crystals pierce through tissue, and upon thawing leave hollow structures, causing damage to the tissue, which affects the integrity and reduces the quality of the tissue for histological examinations.

Cryogenic sprays are safer than the LN method, and therefore are more commonly used in pathology departments to rapidly freeze fresh tissues. However, the method of using cryogenic sprays can vary from one lab to another. For example, especially when the fresh tissue specimen to be frozen is very small, it should be kept cold from the moment when it is frozen with the cryogenic spray; otherwise, the tissue starts to thaw within a few seconds of freezing, Consequently, re‐freezing the tissue after transferring to a freezer for long‐term storage can cause re‐formation of large ice crystals. For these reasons, tissues frozen by cryogenic spray should be kept at freezing temperatures at all times (between freezing and the transport to a freezer). Considering these factors, we have combined the use of cryogenic spray and freezing on the cold aluminum platform into a new technique of freezing fresh tissue, ensuring the best preservation of the tissue. In this new method, the flat surface of the aluminum platform is used as a platform to keep the frozen tissue at below‐freezing temperatures and avoid accidental thawing of the small tissue samples.

RNA degradation, particularly in enzymatically rich samples such as the pancreas, continues to pose a problem for researchers interested in RNA biology (Chu et al., 2002; Leonard et al., 1993). Indeed, some studies showed that better preservation of RNA can be achieved by vapor‐phase liquid nitrogen (VPLN) during long‐term (5‐12 years) storage (Auer et al., 2014). Furthermore, samples stored in −80°C freezers require an additional treatment with RNAlater^®^ or a similar RNA‐stabilizing solution for better preservation of RNA over longer periods, which can protect them even when the samples are thawed and left at room temperature for up to 16 hr (Botling et al., 2009). Nevertheless, most freezing methods yield better quality and quantity of DNA and RNA compared to FFPE samples for use in current approaches such as whole‐genome amplification, whole‐genome sequencing, and cDNA microarray analyses (Mareninov et al., 2013). Also, in fresh‐frozen tissue, proteins are well preserved, including intact enzymatic activity, which is lost in the FFPE specimens due to the crosslinking (von Teichman, Storz, Dettwiler, Moch, & Schraml, 2012). One of the negatives of frozen tissue specimens is that infectious organisms within the tissue may remain viable, so universal precautions are necessary when handling these specimens. In this study, we also demonstrate that delays of sample freezing up to 1 hr had no detrimental effect on the quality or integrity of frozen murine liver and pancreas tissue with the AP technique, as well as the DI and LN freezing techniques.

Thus, the addition of an aluminum platform and tubes pre‐cooled using dry ice, and rapid freezing with cryogenic spray of biological tissue wrapped in aluminum foil, could be simple adaptations that can be rapidly deployed widely, enabling standard bio‐specimen collection. We encourage researchers to use this method for a wider range of tissues.

### Troubleshooting

The “Leidenfrost effect” of liquid nitrogen, which was briefly mentioned above, can negatively affect the rapid freezing of the specimen. This effect occurs when a sample tube at room temperature is inserted into liquid nitrogen, which has a much lower boiling point than the surface temperature of the tube. As a result, rapid boiling of liquid nitrogen at the contact points on the tube forms an insulating vapor layer between the liquid nitrogen and the sample tube, slowing down freezing. This slower freezing can result in excessive ice‐crystal formation in the tissue, increased tissue degradation, and cumulative damaging effects on sample integrity. This boiling effect can be mitigated by pre‐cooling the tube in order to reduce the temperature difference between the tube and the boiling temperature of LN. Additional problems that can arise with the techniques described in this article are listed, along with their potential causes and solutions, in Table 1.

**Table 1:** Troubleshooting Guide for Freezing Fresh Tissues

Problem	Cause	Solution
Leidenfrost effect	Temperature difference between tube and LN	Pre‐cool the empty specimen tubes in dry ice
Dry tissue	Tissue is not stored in enough PBS solution during transport or incubation	Make sure the fresh tissue is completely submerged in PBS solution; increase the volume of PBS in the Petri dish where the fresh tissue is stored
Soft tissue after cryospray freezing	Not aiming the nozzle of the cryospray directly at the fresh tissue or not spraying long enough	Make sure the nozzle of the cryospray is aimed directly to the fresh tissue and sprayed continuously for at least 10 s
Unintended thawing of the frozen tissue during storage in dry ice	Frozen tissue tubes are placed on top of the dry ice and not submerged completely in dry ice	Make sure the frozen tubes are completely submerged and covered by dry ice during the procedure, until the tubes are transferred to a freezer for long‐term storage
Tube cannot be completely submerged in liquid nitrogen	Not having enough liquid nitrogen in the transport Dewar	Make sure there is enough liquid nitrogen in the LN transport Dewar so that the tube can be fully submerged into LN during the freezing. The height of LN in the Dewar should be a minimum of 3 cm. This height is enough for a 0.7‐ml FluidX™ tube to be fully submerged.
Frozen tissue does not fit into 0.7‐ml FluidX™ tube after freezing with AP method	Fresh tissue is too large	Make sure the size of the tissue is appropriate (having a maximum diameter of 4 mm and maximum length of 1 cm). Cut larger fresh tissue into a smaller appropriate size with a scalpel before freezing with cryospray.

### Conflict of Interest Statement

The authors declare that there is no conflict of interest.

### Author Contributions

**Ahmet Imrali**: Conceptualization; data curation; formal analysis; investigation; methodology; resources; writing‐original draft; writing‐review & editing. **Christine S. Hughes**: Data curation; formal analysis; methodology; writing‐review & editing. **Abigail S. Coetzee**: Formal analysis; investigation; methodology; writing‐review & editing. **Francesca R. Delvecchio**: Formal analysis; methodology; writing‐review & editing. **Amina Saad**: Methodology; writing‐review & editing. **Rhiannon Roberts**: Investigation; project administration; writing‐review & editing. **Claude Chelala**: Supervision; visualization; writing‐review & editing. **Jo‐Anne ChinAleong**: Formal analysis; investigation; methodology; project administration; supervision; visualization; writing‐review & editing. **Hemant M. Kocher**: Conceptualization; data curation; formal analysis; funding acquisition; investigation; methodology; project administration; resources; supervision; validation; visualization; writing‐original draft; writing‐review & editing.

## References

[cpet46-bib-0001] Auer, H. , Mobley, J. A. , Ayers, L. W. , Bowen, J. , Chuaqui, R. F. , Johnson, L. A. , … Ramirez, N. C. (2014). The effects of frozen tissue storage conditions on the integrity of RNA and protein. Biotechnic & Histochemistry, 89, 518–528. doi: 10.3109/10520295.2014.904927. 24799092 PMC4213858

[cpet46-bib-0002] Balarajah, V. , Ambily, A. , Ullah, A. Z. D. , Imrali, A. , Dowe, T. , Al‐Sarireh, B. , … Kocher, H. M. (2016). Pancreatic cancer tissue banks: Where are we heading? Future Oncology, 12, 2661–2663. Epub 2016 Aug 2619. doi: 10.2217/fon-2016-0243. 27541064

[cpet46-bib-0003] Bertschinger, P. , & Bollinger, A. (1995). [Backache, claudicatio venosa and chronic diarrhea]. Der Internist, 36, 1179–1181. 8567224

[cpet46-bib-0004] Betsou, F. , Barnes, R. , Burke, T. , Coppola, D. , Desouza, Y. , Eliason, J. , … International Society for Biological and Environmental Repositories (ISBER) Working Group on Biospecimen Science . (2009). Human biospecimen research: Experimental protocol and quality control tools. Cancer Epidemiology, Biomarkers & Prevention, 18, 1017–1025. doi: 10.1158/1055-9965.EPI-08-1231. 19336543

[cpet46-bib-0005] Bonini, P. , Plebani, M. , Ceriotti, F. , & Rubboli, F. (2002). Errors in laboratory medicine. Clinical Chemistry, 48, 691–698. doi: 10.1093/clinchem/48.5.691. 11978595

[cpet46-bib-0006] Botling, J. , Edlund, K. , Segersten, U. , Tahmasebpoor, S. , Engström, M. , Sundström, M. , … Micke, P. (2009). Impact of thawing on RNA integrity and gene expression analysis in fresh frozen tissue. Diagnostic Molecular Pathology, 18, 44–52. doi: 10.1097/PDM.0b013e3181857e92. 19214109

[cpet46-bib-0007] Chu, T.‐Y. , Hwang, K.‐S. , Yu, M.‐H. , Lee, H.‐S. , Lai, H.‐C. , & Liu, J.‐Y. (2002). A research‐based tumor tissue bank of gynecologic oncology: Characteristics of nucleic acids extracted from normal and tumor tissues from different sites. International Journal of Gynecological Cancer, 12, 171–176. doi: 10.1046/j.1525-1438.2002.01085.x. 11975676

[cpet46-bib-0008] Leonard, S. , Logel, J. , Luthman, D. , Casanova, M. , Kirch, D. , & Freedman, R. (1993). Biological stability of mRNA isolated from human postmortem brain collections. Biological Psychiatry, 33, 456–466. doi: 10.1016/0006-3223(93)90174-C. 8098224

[cpet46-bib-0009] Mareninov, S. , De Jesus, J. , Sanchez, D. E. , Kay, A. B. , Wilson, R. W. , Babic, I. , … Yong, W. H. (2013). Lyophilized brain tumor specimens can be used for histologic, nucleic acid, and protein analyses after 1 year of room temperature storage. Journal of Neuro‐Oncology, 113, 365–373. doi: 10.1007/s11060-013-1135-1. 23640138 PMC3886564

[cpet46-bib-0010] Mehta, R. , Baranova, A. , & Birerdinc, A. (2012). Do‐It‐Yourself device for recovery of cryopreserved samples accidentally dropped into cryogenic storage tanks. Journal of Visualized Experiments, e3903. doi: 10.3791/3903. 22617806 PMC3466944

[cpet46-bib-0011] Rivas Leonel, E. C. , Lucci, C. M. , & Amorim, C. A. (2019). Cryopreservation of human ovarian tissue: A review. Transfusion Medicine and Hemotherapy, 46, 173–181. doi: 10.1159/000499054. 31244585 PMC6558345

[cpet46-bib-0012] Shabihkhani, M. , Lucey, G. M. , Wei, B. , Mareninov, S. , Lou, J. J. , Vinters, H. V. , … Yong, W. H. (2014). The procurement, storage, and quality assurance of frozen blood and tissue biospecimens in pathology, biorepository, and biobank settings. Clinical Biochemistry, 47, 258–266. doi: 10.1016/j.clinbiochem.2014.01.002. 24424103 PMC3982909

[cpet46-bib-0013] Tang, W. , Hu, Z. , Muallem, H. , & Gulley, M. L. (2012). Quality assurance of RNA expression profiling in clinical laboratories. The Journal of Molecular Diagnostics, 14, 1–11. doi: 10.1016/j.jmoldx.2011.09.003. 22020152 PMC3338342

[cpet46-bib-0014] Von Teichman, A. , Storz, M. , Dettwiler, S. , Moch, H. , & Schraml, P. (2012). Whole genome and transcriptome amplification: Practicable tools for sustainable tissue biobanking? Virchows Archiv, 461, 571–580. doi: 10.1007/s00428-012-1315-y. 23007645

[cpet46-bib-0015] https://npin.cdc.gov/publication/updated-us-public-health-service-guidelines-management-occupational-exposures-human.

[cpet46-bib-0016] https://www.thepancreastissuebank.org/.

